# 

**Published:** 1840

**Authors:** 


### WESTERN JOURNAL

### OF

### MEDICINE AND SURGERY.

### COLLABORATORS.

EDWARD H. BARTON, M. D. *Professor of the Theory and Practice of Medicine, in the Medical College of Louisiana.*

WILLIAM J. BARBEE, M. D. *Georgetown, Ky.*

GEORGE W. BAYLESS, M. D. *Dissector for the Pathological Cabinet of the Louisville Medical Institute.*

A. H. BUCHANAN, M. D. *Columbia, Tennessee.*

CHARLES CALDWELL, M. D. *Professor of the Institutes of Medicine and Medical Jurisprudence, in the Louisville Medical Institute.*

SAMUEL A. CARTWRIGHT, M D. *Mississippi.*

A. CLAPP, M. D. *New Albany, Indiana.*

JEDEDIAH COBB, M. D. *Professor of Anatomy, in the Louisville Medical Institute.*

JOHN ESTEN COOKE, M. D. *Professor of the Theory and Practice of Medicine, in the Louisville Medical Institute.*

SAMUEL D. GROSS, M. D. *Professor of Surgery in the Louisville Medical Institute.*

JOHN HARDIN, M. D. *Munfordsville, Ky.*

JOHN P. HARRISON, M. D. *late Professor of Materia Medica, in the Cincinnati College.*

JOHN F. HENRY, M. D. *Bloomington, Illinois.*

SAMUEL P. HILDRETH, M. D. *Marietta, Ohio.*

SAMUEL HOGG, M. D. *Nashville, Tennessee.*

M. Z. KREIDER, M. D., *Lancaster, Ohio.*

MOSES L LINTON, M. D. *Springfield, Kentucky.*

HENRY MILLER, M. D. *Professor of Obstetrics and the Diseases of Women and Children, in the Louisville Medical Institute.*

JOHN W. MONETTE, M. D. *Washington, Mississsippi.*

HENRY PERRINE, M. D. *Florida.*

SAMUEL B. RICHARDSON, M. D. *Louisville, Ky.*

JOHN L. RIDDELL, M. D. *Professor of Chemistry in the Medical College of Louisiana.*

LANDON C. RIVES, M. D. *late Professor of Obstetrics, in the Medical Department of the Cincinnati College.*

CHARLES W. SHORT, M. D. *Professor of Materia Medica, in the Louisville Medical Institute.*

G. TROOST, M. D. *Professor of Chemistry and Mineralogy, in the University of Nashville.*

AMASA L. TROWBRIDGE, M. D. *Professor of Surgery, Willoughby University, Ohio.*

JOHN A. WARDER, M. D. *Cincinnati, Ohio.*

WILLIAM WOOD, M. D. *Cincinnati, Ohio.*

### TO READERS AND CORRESPONDENTS.

Communications have been received from Drs. Hogg, Cartwright, Dickinson, Lawrie, Searcy, Bouchelle, Monette, and Caldwell.

The following works have been received:

Fifth Geological Report to the Twenty-third General Assembly of Tennessee, by G. Troost, M. D., Geologist to the State; Professor of Chemistry, Mineralogy and Geology in the Nashville University; and Member of the Geological Societies of France and Pennsylvania, of the American Philosophical Society, and of the Academy of Natural Sciences, Philadelphia. (From the Author.)

A Lecture Introductory to the Course of Surgery in the Jefferson Medical College, Philadelphia, for the Session of 1839—'40. By Joseph Pancoast, M. D., Professor of the Institutes and Practice of Surgery in Jefferson Medical College; one of the Surgeons to the Philadelphia Hospital; Fellow of the College of Physicians, &c. &c. (From the Author.

A system of Medical Etiquette, Rules and Regulations, as adopted by the Medical Association of North Eastern Kentucky. (From Dr. Duke.)

Thirteenth Annual Report of the Trustees of the Ohio Asylum for the Education of the Deaf and Dumb, for the year 1839. (From H. N. Hubbell, Esq.)

Introductory Lecture by Willis Baxley, M. D., Professor of Anatomy and Physiology in the University of Maryland, Baltimore 1839. (From the Author.)

The Salt Sulphur Springs of Monroe County, Va. By Thomas D. Mutter, M. D., Lecturer on Surgery, Corresponding Member of the New York Medical and Surgical Society; Fellow of the College of

Physicians of Philadelphia, &c. &c. Philadelphia, 1840. (From the Author.)

The American Journal of the Medical Sciences for February, 1840. (In exchange.)

The American Medical Library and Intelligencer, from February to June. (In exchage.)

The Maryland Medical and Surgical Journal. (In exchange.)

The Medical Examiner, (In exchange.)

The Homœopathic Examiner for January and February. (In exchange.)

The New York Journal of Medicine and Surgery. (In exchange.)

The Boston Medical and Surgical Journal. (In Exchange.)

☞The June number, which will complete the first volume of the Western Journal of Medicine and Surgery, will be put to press immediately, and issued without loss of time.

THE

### WESTERN JOURNAL

OF

### MEDICINE AND SURGERY,

Is published monthly by Prentice & Weissinger, corner of Main and Fifth-streets, Louisville, and edited by Prof. Drake and Prof. Yandell, assisted by their colleagues, and a number of gentlemen, not members of the school. Each number contains 80 pages, making two volumes a year, of about 500 pages. The first volume will be completed with the number for the present month—June. Contributions to its pages, by the physicians of the Valley of the Mississippi, are respectfully solicited. The subscription to this, the only medical journal now publishing west of the mountains, is five dollars *per annum.*

PRENTICE & WEISSINGER.

*Louisville, June,* 1840.

